# Exogenous Microorganisms Promote Moss Biocrust Growth by Regulating the Microbial Metabolic Pathway in Artificial Laboratory Cultivation

**DOI:** 10.3389/fmicb.2022.819888

**Published:** 2022-03-02

**Authors:** Chang Tian, Heming Wang, Shufang Wu, Chongfeng Bu, Xueqiang Bai, Yahong Li, Kadambot H. M. Siddique

**Affiliations:** ^1^Institute of Soil and Water Conservation, CAS and MWR, Yangling, China; ^2^University of Chinese Academy of Sciences, Beijing, China; ^3^College of Water Resources and Architectural Engineering, Northwest A&F University, Yangling, China; ^4^Institute of Soil and Water Conservation, Northwest A&F University, Yangling, China; ^5^The UWA Institute of Agriculture and School of Agriculture & Environment, The University of Western Australia, Perth, WA, Australia

**Keywords:** moss crusts, exogenous additives, PICRUSt2, microbial function, artificial laboratory cultivation, high-throughput sequencing

## Abstract

Moss-dominated biocrusts (moss crusts) are a feasible approach for the ecological restoration of drylands, but difficulty obtaining inoculum severely limits the progress of large-scale field applications. Exogenous microorganisms could improve moss growth and be conducive to moss inoculum propagation. In this study, we investigated the growth-promoting effects and potential mechanisms of exogenous microorganism additives on moss crusts. We used an incubator study to examine the effects of inoculation by heterotrophic microorganisms (*Streptomyces pactum*, *Bacillus megaterium*) and autotrophic microorganisms (*Chlorella vulgaris*, *Microcoleus vaginatus*) combined with *Artemisia sphaerocephala* gum on the growth of *Bryum argenteum*, the dominant moss crusts species in sandy deserts. Amplicon sequencing (16S and 18S rRNA) and PICRUSt2 were used to illustrate the microbial community structure and potential function in the optimal treatment at different developmental stages. Our results showed that exogenous microorganisms significantly promoted moss growth and increased aboveground biomass. After 30 days of cultivation, the *Streptomyces pactum* (1 g kg^–1^ substrate) + *Chlorella vulgaris* (3.33 L m^–2^) treatment presented optimal moss coverage, height, and density of 97.14%, 28.31 mm, and 2.28 g cm^–2^, respectively. The best-performing treatment had a higher relative abundance of Streptophyta—involved in moss growth—than the control. The control had significantly higher soil organic carbon than the best-performing treatment on day 30. Exogenous microorganisms improved eukaryotic community diversity and richness and may enhance soil microbial functional and metabolic diversity, such as growth and reproduction, carbon fixation, and cellulose and lignin decomposition, based on functional predictions. In summary, we identified the growth-promoting mechanisms of exogenous additives, providing a valuable reference for optimizing propagation technology for moss inoculum.

## Introduction

Most dryland ecosystems are regulated and stressed by abiotic factors, especially water, so it is hard for drylands to support the growth of abundant and continuously distributed vascular plants (Li et al., 2009). The patchy distribution of vascular plants provides a suitable ecological niche for biological soil crusts (biocrusts) colonization, covering 40% of the dryland surface area (Li et al., 2009; Lan et al., 2012). Biocrusts are a community of cyanobacteria, algae, lichens, bryophytes, and other microorganisms integrated with topsoil particles (Eldridge and Greene, 1994; Belnap et al., 2016) that provide several ecosystem services, including nitrogen and carbon fixation (Beymer and Klopatek, 1991; Billings et al., 2003), soil stability (Zhang et al., 2007), microhabitats for other organisms (Liu et al., 2013), and important interactions with vascular plants (Eldridge and Greene, 1994; Chaudhary et al., 2009; Li et al., 2009). Therefore, biocrusts protection and cultivation are essential for dryland restoration. However, biocrusts are sensitive to all kinds of disturbances that can take decades or centuries to recover (Brotherson et al., 1983; Belnap, 2003; Yüksek, 2009; Evans and Johansen, 2010). If drylands rely on natural recovery only, there will be a lack of stable and fully functional biocrusts for many years after disturbance. Thus, manual intervention, such as field inoculation, is vital for dryland restoration.

In recent years, field-inoculation restoration experiments have made remarkable progress, indicating that collecting field biocrusts as inoculum is effective for dryland restoration (Belnap, 1996; Chiquoine et al., 2016; Li et al., 2018; Schaub et al., 2019). However, it causes artificial disturbances, damaging the local ecological environment. Using artificially cultivated instead of field-collected biocrusts can solve this problem. Thus, many scholars have investigated methods to cultivate moss crusts artificially, the biocrusts with moss as the dominant species, which is also the advanced stage of biocrusts (Chamizo et al., 2013; Garibotti et al., 2018), with some success (Bu et al., 2013; Chamizo et al., 2013; Li et al., 2018; Schaub et al., 2019). Zhao et al. (2018) and Pereira et al. (2021) obtained many different species of mosses by culturing gametophyte fragments *in vitro*. Chen et al. (2009) developed moss crusts using three inoculation methods (moss spores, moss fragments, and ground moss crusts), reporting that the ground moss crusts had the most potential. These incubator studies have successfully cultivated moss crusts using different methods, confirming the feasibility of moss inoculum propagation. However, the current efficiency of their cultivation cannot meet the needs of dryland restoration, and thus high-efficiency propagation technology for moss inoculum is urgently needed.

It is important to clarify the factors affecting moss growth to improve propagation efficiency. Many studies have shown that low-level light, temperature, and nutrient solution concentration promote moss growth (Wu et al., 2001; Bu et al., 2011; Zhang, 2012; Ding et al., 2015; Yang et al., 2015). Watering frequency, moss species, substrate and binder type, and inoculation method significantly affect the morphological indexes of moss (Jia et al., 2012; Antoninka et al., 2016; Yang et al., 2016; Bu et al., 2018; Blankenship et al., 2020). Ju et al. (2019) and Wang et al. (2019) reported that adding *Chlorella vulgaris* and *Bacillus mucilaginosus* to the substrate significantly improves moss crusts coverage and thickness. Nevertheless, the growth-promoting mechanism of these exogenous microorganisms during artificial moss crusts cultivation is unknown, limiting the development of moss inoculum propagation.

A symbiotic system of heterotrophic and autotrophic microorganisms can promote microbial community stability and diversity, which is more conducive to stable and sustainable culture than a single microorganism culture system (Feng et al., 2021; Zhang A. L. et al., 2021) and thus may improve the artificial cultivation efficiency of moss crusts. Among heterotrophic microorganisms, *Bacillus megaterium* promotes the dissolution of insoluble phosphate in soil and plant growth (Jahil and Kamal, 2021); *Actinomycetes* is central to soil microbial communities and *Streptomyces* produces substances that help plants resist harsh environments (Zhao et al., 2012; Cao et al., 2016). Among autotrophic microorganisms, *Microcoleus vaginatus*, a cyanobacteria species, is the dominant biocrusts species and plays a key role in promoting biocrusts formation and development (Jing et al., 2017); *Chlorella vulgaris* increases the amount of soil nutrients through physiological metabolism, such as photosynthesis (Odgerel and Tserendulam, 2017; Alobwede et al., 2019). *Artemisia sphaerocephala* Krasch. gum (ASKG)—a natural binder extracted from *Artemisia sphaerocephala* Krasch. and the major species at our moss collection site—significantly increases the compressive strength of moss crusts and provides a relatively stable microenvironment (Liu et al., 2016). Based on the above, the combined impact of heterotrophic microorganisms, autotrophic microorganisms, and binder on moss crusts growth should be explored.

*Bryum argenteum*, the dominant moss crusts species collected from the Mu Us Sandland in China, was cultivated artificially in an incubator. A three-factor, two-level orthogonal experiment was undertaken to reveal the effectiveness of gum (added or not added), heterotrophic microorganisms (*Streptomyces pactum*, *Bacillus megaterium*), and autotrophic microorganisms (*Microcoleus vaginatus, Chlorella vulgaris*) on moss growth. The study explored (1) the exogenous additive combination of best performing treatment for promoting moss crusts growth; (2) changes in microbial community composition under the optimal treatment with exogenous additives compared to a control; (3) the microbial functional prediction under the optimal treatment; (4) the growth-promoting mechanisms of the optimal treatment on moss crusts. We aimed to improve the cultivation technology of moss crusts and provide a theoretical basis for large-scale engineering proliferation of moss inoculum.

## Materials and Methods

### Moss Crusts Inoculum and Microbial Inoculum Preparation

**FIGURE 1:**
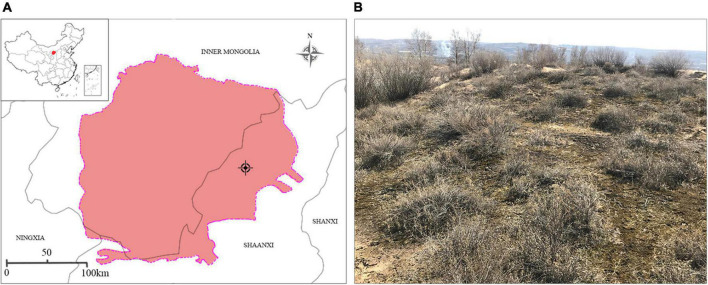
**(A)** Location and **(B)** landscape of the site where moss crusts and the substrate were collected.

The moss crusts and substrate used for cultivation were collected from Yuyang District (109°60′ E, 38°53′ N), Yulin City, Shaanxi Province, China (Figure 1A). This region has an average annual precipitation of 405 mm (mainly occurring between June and September) and a mean annual temperature of 8.3°C (Mo and Ren, 2009). The sample plots were fixed and semi-fixed sand dunes close to the southeast edge of the Mu Us Sandland. The primary shrub species, distributed mainly in clusters, include *Caragana korshinskii*, *Artemisia desertorum*, and *Salix psammophila*. Herbaceous plants are relatively scattered, including *Psammochloa villosa* and *Heteropappus hispidus* (Figure 1B).

The well-developed moss crusts (about 1 cm thick) were collected with a shovel, transported to the laboratory, and air-dried. The dominant moss crusts species was *Bryum argenteum*, followed by *Bryum rutilans* Brid and *Gymnostomum calcareum* Nees et Hornsch. Conspicuous impurities, such as livestock manure, plant litter, roots, and stones, were removed manually. The moss crusts were crushed into stem and leaf fragments (1–3 mm) using a plant sample pulverizer (IZARA-2500C, Jinhua, China) and mixed well for moss inoculum cultivation. The soil used as the substrate in the experiment was collected from the subsoil (5–20 cm depth) of the moss crusts and sieved (2.0 mm mesh) after air-drying in the laboratory. The sifted sandy soil was dried and sterilized at 108°C for 80 min.

Strains of *Chlorella vulgaris* (FACHB-8) and *Microcoleus vaginatus* (FACHB-2003), cultivated in BG11 medium, were purchased from Freshwater Algae Culture Collection at the Institute of Hydrobiology, Wuhan, China.^1^ The spore powder of *Bacillus megaterium* (BM22), provided by the College of Life Sciences, Northwest A&F University, Yangling Shaanxi, China, had a spore density of 1 × 10^10^ CFU g^–1^. The spore powder of *Streptomyces pactum* (Genbank: MH542148), provided by the Laboratory of Microbial Resources in College of Resources and Environment, Northwest A&F University, Yangling Shaanxi, China, had a spore density of 6.36 × 10^9^ CFU g^–1^.

### Experimental Design and Process

The orthogonal experimental design uses orthogonal tables to understand the complete experiment through part experiments and identifies the main factors with significant effect on the indexes to determine the best combination of factors and levels (Xu, 1992; Hao and Yu, 2005). We used an orthogonal experiment to test the effectiveness of adding moss fragments and different combinations of autotrophic microorganisms, heterotrophic microorganisms, and gum on moss growth. There were four treatments, each with three replicates: (1) gum + *Bacillus megaterium* + *Chlorella vulgaris* (ABC), (2) gum + *Streptomyces pactum* + *Microcoleus vaginatus* (ASM), (3) *Bacillus megaterium* + *Microcoleus vaginatus* (BM), (4) *Streptomyces pactum* + *Chlorella vulgaris* (SC), and (5) control (no gum or exogenous microorganisms) (CK) (Table 1).

**TABLE 1:** Description of the four treatment combinations for *Artemisia sphaerocephala* gum additive, heterotrophic microorganisms, and autotrophic microorganisms, and the control used in the incubator study.

Treatment name	*Artemisia sphaerocephala* gum	Heterotrophic microorganisms	Autotrophic microorganisms
ABC	Gum	*Bacillus megaterium*	*Chlorella vulgaris*
ASM	Gum	*Streptomyces pactum*	*Microcoleus vaginatus*
BM	No gum	*Bacillus megaterium*	*Microcoleus vaginatus*
SC	No gum	*Streptomyces pactum*	*Chlorella vulgaris*
CK (control)	No gum	No exogenous microorganisms	

The indoor propagation experiment was carried out at the Engineering Center of Institute of Soil and Water Conservation Laboratory, CAS & MWR. The incubator was set to 20°C with a 12 h:12 h (light:dark) photoperiod (light at 6000 lux and 75% air humidity), consistent with previous studies (Garcia-Pichel and Pringault, 2001; Li et al., 2009).

The moss inoculum was cultivated in the culture boxes. Each replicate contained two different-sized culture boxes. Samples for analyzing soil chemical properties and microbial community structure were taken from the square boxes (17 cm long, 17 cm wide, 12 cm high), and morphological indexes of moss crusts were measured in the rectangular boxes (22 cm long, 17 cm wide, 12 cm high) to avoid confusion. A piece of felt cloth—disinfected with potassium permanganate diluent—covered the bottom of each sterilized box to prevent the substrate from leaking through the water drainage holes. Next, 5 cm of sand mixed with *Streptomyces pactum* (1g kg^–1^ substrate) (Guo et al., 2020) or *Bacillus megaterium* (1g kg^–1^ substrate) (Liu et al., 2012) was added to each box, and the surface of the substrate was flattened before distributing the moss inoculum evenly (1,020 g m^–2^) (Bu et al., 2018). For the gum treatments, gum (6 g m^–2^) (Liu et al., 2016) was mixed with the moss inoculum. After inoculation, all treatments were sprayed with 200 mL water to ensure that the mass gum concentration reached 0.2%. A suspension of *Microcoleus vaginatus* (3.33 L m^–2^) (Xie et al., 2008; Jing et al., 2017) or *Chlorella vulgaris* (3.33 L m^–2^) (Zhang H. J. et al., 2021) was sprayed evenly on the sample after inoculation.

### Monitoring and Maintenance

The moss inoculation day was taken as day 0. During cultivation, Hoagland nutrient solution (2.1 L m^–2^) was sprayed every 6 days (Bu et al., 2018), and morphological indexes for the moss crusts (plant height, coverage, and density) were measured every 7 days. The cultivation goal is to promote moss growth, so the best-performing treatment was confirmed based on morphological indexes of moss. We performed amplicon sequencing and measured soil chemical properties in the best-performing treatment to explore the growth-promoting mechanisms of exogenous microorganisms at day 1, day 15, and day 30. When the moss yellowed, the experiment ended (day 30). The heights of 20 random mosses were measured with an electronic vernier caliper, with the average value taken as plant height. Moss density was measured in five 2.5 cm × 2.5 cm grids on the diagonals of each plot. Moss crusts coverage was measured using a point sampling frame (0.5 cm × 0.5 cm grid) (Li et al., 2010). Soil organic carbon (SOC) was determined using the dichromate oxidation method (Walkley and Black, 1934). Total nitrogen (TN) was determined using a Kjeldahl Apparatus Nitrogen Analyzer (FOSS 2200, Jiangsu, China).

### Soil Microbial Community Analysis

Soil microbial diversity and community composition in the moss crusts were determined using DNA extraction and Illumina sequencing of 16S and 18S genes (Xiao and Veste, 2017; Zhang et al., 2018). The moss sequences (e.g., chloroplasts, mitochondria, and occasional contaminated leaves) were excluded. For each sample, total genomic DNA was extracted from 0.5 g soil using a Mo Bio Power Soil DNA Isolation Kit (Mo Bio Laboratories, Carlsbad, CA, United States). Soil prokaryotic communities were assessed by sequencing the 16S V4-V5 region of the RNA operon with the broad-spectrum primer set 515F (5′-GTGCCAGCMGCCGCGG-3′) and 909R (5′-CCCCGYCAATTCMTTTRAGT-3′) (Narihiro and Sekiguchi, 2015). Soil eukaryotic communities were assessed by sequencing the 18S V4 region of the RNA operon with the broad-spectrum primer set 573 (5′-CGCGGTAATTCCAGCTCCA-3′) and 951 (5′- TTGGYRAATGCTTTCGC-3′) (Mangot et al., 2013). DNA samples were amplified, purified, and sequenced on the Illumina MiSeq 250 PE platform at Allwegene Company, Beijing.

The raw sequences of prokaryotic and eukaryotic reads were trimmed using Mothur, retaining sequences with the following three criteria: (1) precise primers and barcodes; (2) quality score > 30; (3) length > 200 bp. The Ribosomal Database Project (RDP) classifier tool (Wang et al., 2007) was used to classify all sequences into different taxonomic groups. Qualified reads were separated using sample-specific barcode sequences and trimmed with Illumina Analysis Pipeline Version 2.6. The dataset was then analyzed using QIIME2. The sequences were clustered into operational taxonomic units (OTUs) at a similarity level of 97% (Colwell and Coddington, 1994). The RDP Classifier tool was used to classify all sequences into different taxonomic groups, with a 70% confidence threshold (Cole et al., 2009).

Based on the method of Bokulich et al. (2013), some rare OTUs (< 0.001% of total sequences), often associated with spurious reads, were removed before the final analysis. There were differences in the number of clean tags for each sample (Supplementary Table 1). To analyze the alpha diversity, the number of sequences for each sample should be the same. Therefore, we rarefied 21,084 (for the 16S rRNA gene) and 32,556 (for the 18S rRNA gene) final tags of randomly selected clean tags per sample to correct for differences in sequencing depth. The average percentage of the final tags used in the downstream analysis was 43.0% (26.8–73.2%) for the 16S rRNA gene and 39.3% (8.5–90.3%) for the 18S gene (Supplementary Table 1). The rarefaction curves (Supplementary Figure 1) for all samples represented good sampling depth. The Basic Local Alignment Search Tool (BLAST) (V2.6.0) was used to classify all sequences into taxonomic groups based on the Silva138 SSU database (Quast et al., 2013) for the 16S rRNA gene and the Unite 7.2 database^2^ for the 18S rRNA gene. The raw sequencing data have been deposited into the NCBI Sequence Read Archive database under Accession Number PRJNA669273.

### Predicted Gene Expression Analysis

PICRUSt2 is software for predicting functional abundance based on marker gene sequences (Dong et al., 2018). The OTUs information obtained through sequencing and classification are inputted into to compare with sequenced species in the microbial genome database. PICRUSt2 annotates OTUs as the corresponding species, and outputs the functional types and corresponding functional abundance according to the abundance of OTUs (Zhang et al., 2019; Douglas et al., 2020). The accuracy of PICRUSt2 has improved due to increased taxonomic diversity coverage of the reference genome database (Langille et al., 2013). Default gene banks, such as Kyoto Encyclopedia of Genes and Genomes (KEGG Orthologs, KO) and Enzyme Commission numbers (EC no.), were used to support functional potential.

### Data Analyses

We used one-way ANOVA and Duncan’s Multiple Range Test to examine differences in moss growth indices among the four treatments and control at four growth points (7, 15, 22, and 30 days). Student’s *t*-tests were used to determine differences in soil organic C and total N between the optimal treatment and the control. Pearson’s correlation analysis was then used to examine potential relationships among moss crusts growth indices, bacterial abundance, and soil nutrients. We used permutational multivariate analysis of variance with the PERMANOVA + for PRIMER (V6) statistical package (Anderson, 2014) and the Bray-Curtis similarity matrix to examine potential differences in prokaryote and eukaryote community composition in relation to time and treatment and their interaction. The first stratum of the analysis considered time; the second, nested within time, considered treatment effects and their interaction with time. The molecular analyses focused on assessing differences between the best-performing treatment and the control. The soil microbial diversity data of the optimal treatment and the control treatment were analyzed using “phyloseq” packages in R (version 4.0.2) for taxonomic composition and alpha diversity (observed OTUs and Chao1) (McMurdie and Holmes, 2013) on days 1, 15, and 30. Differences in alpha diversity and community composition between the optimal treatment and the control were evaluated with student’s *t*-tests using SPSS Statistics software (IBM Corporation, NY, United States). Figures were constructed using Origin 2017 and ggplot2 in R (version 4.0.2).

## Results

### Moss Growth and Soil Chemistry

**FIGURE 2:**
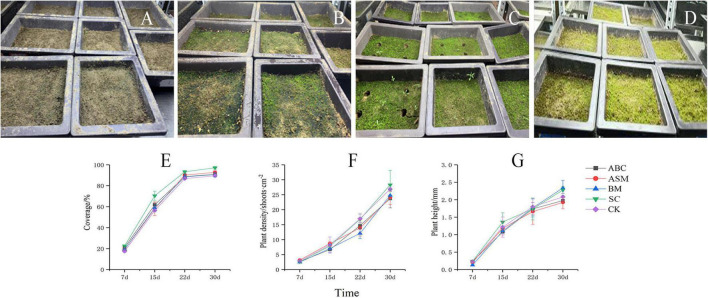
Moss growth status in the *Streptomyces pactum*–*Chlorella vulgaris* treatment without gum at **(A)** 7, **(B)** 15, **(C)** 22, and **(D)** 30 days. Changes in moss **(E)** cover, **(F)** density, and **(G)** height over 30 days in four treatments and the control.

Overall, exogenous microorganisms promote moss biocrusts growth. For example, compared with CK at day 30, microbial addition with (ABC, ASM) and without *Artemisia sphaerocephala* gum (BM, SC) increased moss cover in the crusts (Figure 2E), more so in SC (Figure 2F; one-way ANOVA: *P* < 0.05). SC had greater moss density than CK (Figure 2F), and SC and BM had greater moss height than CK (Figure 2G). SC also had greater moss growth indexes (e.g., coverage, density, and height) than the other treatments, suggesting that those mosses performed best for growth (Figures 2A–D). For example, SC had significantly more moss coverage on day 30 than the other treatments (one-way ANOVA: *P* < 0.05). At the same time, moss density increased by 19.30, 18.25, and 14.25%, and moss height increased by 15.74, 18.75, and 1.79%, respectively, in SC compared to ABC, ASM, and BM. Total nitrogen slightly differed between the best-performing treatment (SC) and CK (Supplementary Table 2). CK had significantly higher SOC than SC on day 30 (Supplementary Table 3; one-way ANOVA: *P* < 0.05).

### Microbial Community Structure in the Best-Performing Treatment

**FIGURE 3:**
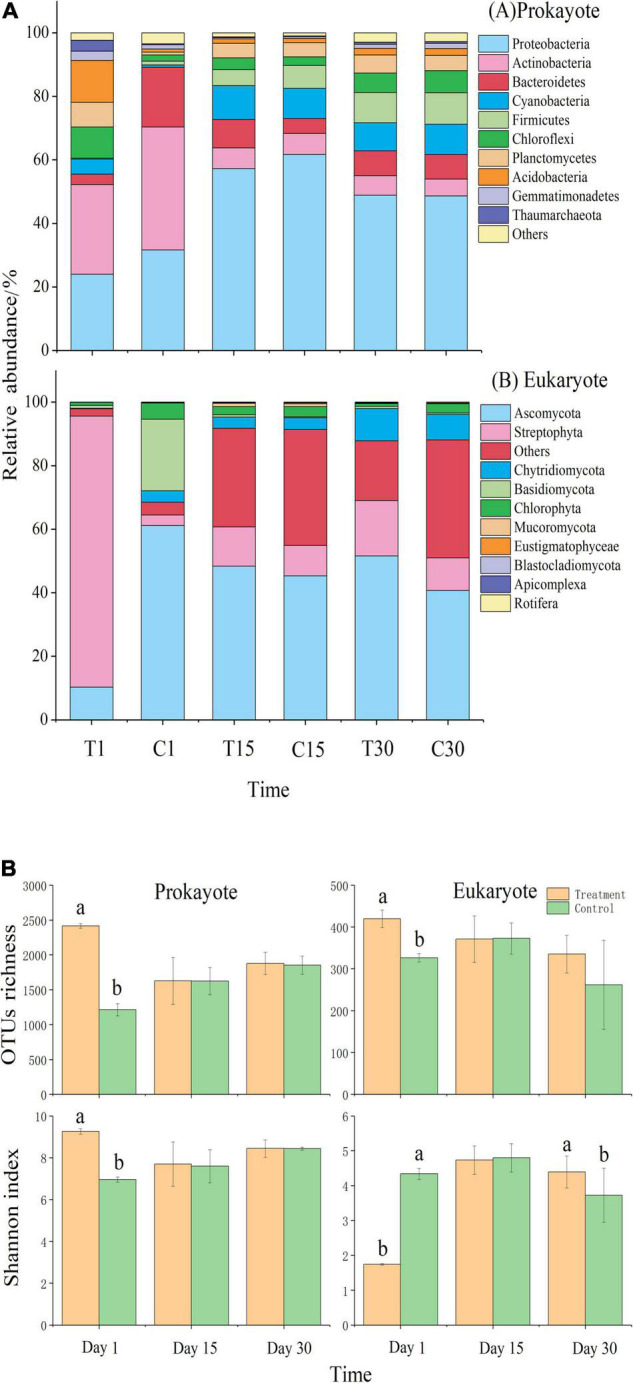
**(A)** Changes in the relative abundance of the main microbial phyla during moss development for (a) prokaryotes and (b) eukaryotes in the best-performing treatment and control; **(B)** Mean (± SE) α-diversity (OTUs richness) and microbial abundance Chao1 index) of prokaryotes and eukaryotes in relation to the treatment (best-performing treatment, control) and time. Significant treatment effects (*P* < 0.05) are shown with different subscripts. T1: treatment on day 1; T15: treatment on day 15; T30: treatment on day 30; C1: control on day 1; C15: control on day 15; C30: control on day.

The addition of exogenous microbes—*Streptomyces pactum* and *Chlorella vulgaris*—altered the microbial community structure of prokaryotes and eukaryotes at each sampling time [PERMANOVA: Pseudo *F*_2_,_6_ = 5.57–5.89; *P* (permutated) = 0.001]. For example, Proteobacteria, Actinobacteria, Streptophyta, and Ascomycota dominated in the best-performing treatment, while Proteobacteria, Actinobacteria, and Ascomycota dominated in CK (abundance > 5% for phyla at all developmental stages) (Figure 3A and Supplementary Table 4). There was no significant difference in prokaryotic composition between treatment and control on days 15 and 30. On day 30, the best-performing treatment had a significantly higher Shannon index than CK (Figure 3B and Supplementary Table 5; one-way ANOVA: *P* < 0.05). The eukaryotic OTUs richness in the best-performing treatment increased by 21.63% compared with CK.

### Prediction of Microbial Metabolic Pathways in the Best-Performing Treatment

**FIGURE 4:**
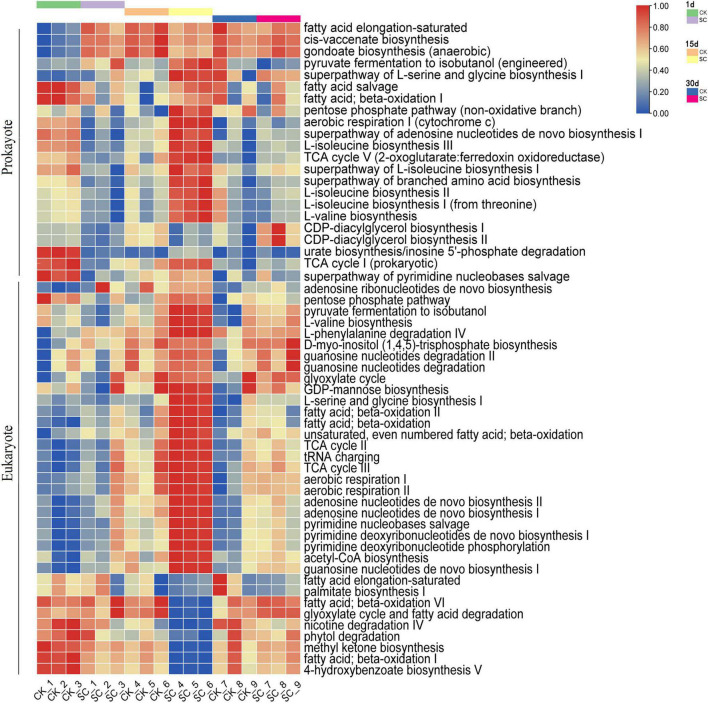
PICRUSt2 function prediction results for metabolic pathways with the relative abundance >0.6% for prokaryotes and >1.0% for eukaryotes in the best-performing treatment and control during moss development.

Analyses of function-related differences between the best-performing treatment (SC) and CK focused on metabolic pathways, with > 0.6% prokaryotes and > 1.0% eukaryotes contributing to significant differences (Figure 4). On day 1, prokaryotes had significantly more metabolic pathways in CK than SC (*P* < 0.05) (Supplementary Table 6). On day 15, SC had significantly more metabolic pathways (16 for prokaryotes and 15 for eukaryotes) than CK (five for prokaryotes and six for eukaryotes) (*P* < 0.05) (Supplementary Table 6), indicating that exogenous microorganisms may enhance the metabolic intensity of the microbial community. We also found that the metabolic pathways were dominated by amino acid synthesis and cell growth in prokaryotes and nucleotide synthesis, fatty acid oxidation, and reproduction in eukaryotes.

**FIGURE 5:**
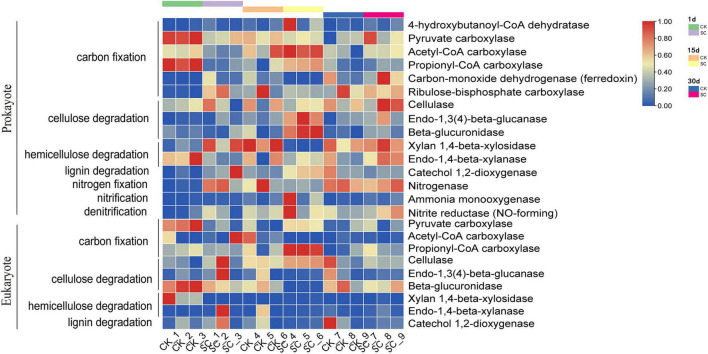
PICRUSt2 function prediction results for the relative abundance of enzymes during moss development for prokaryotes and eukaryotes in the best-performing treatment and control.

PICRUSt2 was used to identify the functional characteristics of soil microbes related to element cycling. We analyzed several key enzymes involved in carbon and nitrogen cycling. For soil prokaryotes, the dominant genes were related to cellulose decomposition and the anaerobic acetyl-CoA and 3-hydroxypropionic acid pathways in carbon fixation. For soil eukaryotes, the dominant genes were related to cellulose decomposition and the oxaloacetate and 3-hydroxypropionic acid pathways in carbon fixation, with comparatively low abundance for other functions (Figure 5 and Supplementary Table 7). On day 1, CK had significantly higher relative abundances for six enzymes than SC, while SC only had two enzymes with significantly higher relative abundances than CK. On day 15, SC had significantly higher relative abundances of six enzymes than CK, while CK only had one enzyme with significantly higher relative abundance than SC, indicating that SC had higher activity of enzymes involved in the carbon and nitrogen cycles than CK. Moreover, on day 15, SC had higher activities of propionyl-CoA carboxylase (carbon fixation), cellulase, endo-1,3(4)- β-glucanase, β-glucuronidase (cellulose decomposition), and catechol 1,2-dioxygenase (lignin decomposition) than CK (Supplementary Table 7).

### Linkages Among Moss Growth, Soil Chemistry and Microbial Indices

Pearson’s correlation analysis showed that the relative abundance of Actinobacteria negatively correlated with most indexes, especially soil TN and organic matter. The Shannon index of eukaryotes negatively correlated with the α diversity index of prokaryotes and positively correlated with soil TN and organic matter. Soil TN and organic matter positively correlated with the moss indexes (Supplementary Figure 2).

## Discussion

Our study showed that adding microbes to the soil improved moss growth irrespective of using gum additive. Furthermore, the treatments differed, and the addition of *Streptomyces pactum* and *Chlorella vulgaris* (best-performing treatment) increased moss cover most. The microbial assemblage also differed between the control and the best-performing treatment, which had greater eukaryotic richness and diversity and some subtle changes in microbial composition with increasing moss growth. Overall, our results suggest that adding exogenous microbes enhances moss coverage and could be a useful technique for propagating moss inoculum.

### Characteristics and Driving Factors of Artificial Moss Growth

The best-performing treatment—the addition of *Streptomyces pactum* and *Chlorella vulgaris*—produced the best growth, in terms of coverage, density, and height (Figures 2E–G). Similar results have been reported on different plants (other than moss), after identifying that *Chlorella vulgaris* functions as a biological fertilizer, enhancing the growth of industrial crops such as *Hibiscus esculentus* (Agwa et al., 2017), *Zea mays* L. (Shaaban, 2001), and Chinese chives (Shaaban, 2001; Agwa et al., 2017; Kim et al., 2018). Similarly, *Streptomyces pactum* produces substances such as hormones (e.g., indole acetic acid and 1-aminocyclopropane-1-carboxylate), iron cells, and metabolites (e.g., antibiotics, organic acids, amino acids, vitamins, and enzymes) that stimulate plant growth (Cao et al., 2016). In addition, the branched mycelia of *Streptomyces pactum* produce a variety of extracellular hydrolases (Zhao et al., 2012) that stimulate root growth and improve nutrient uptake in species such as *Lolium perenne* L. (Cao et al., 2016) and *Panax quinquefolius* L. (Zhang et al., 2013; Cao et al., 2016).

According to our results, the best-performing treatment slightly altered microbial composition. For example, exogenous microbes significantly increased the relative abundance of Streptophyta (Figure 3A). We speculated that the best-performing treatment had many protonemata (juvenile vegetative stage after spore germination of moss), eventually growing into moss and improving its coverage and density. Our results also showed that the best-performing treatment had significantly higher eukaryotic diversity than the control on day 30 (Figure 3B), evidenced by increased eukaryote reproduction (Figure 4 and Supplementary Table 6). Meanwhile, the microbial composition also changed with time after inoculation. For example, the relative abundance of Ascomycota increased in the best-performing treatment (Figure 3A), with greater abundance on days 22 and 30 than the control. The mycelium of Ascomycota stabilized the topsoil, creating more resistance to disturbance and increasing soil water- and nutrition-holding capacity (Zhao et al., 2011). Moreover, Ascomycota are the main functional group of C degradation and cooperatively degrade C substrates (i.e., lignin) for moss growth and development (Zhao et al., 2020).

The control had significantly higher SOC than the best-performing treatment on day 30 (Supplementary Table 3), possibly because soil microbial biomass increases when exogenous microorganisms enter the soil. More extracellular enzymes secreted by the soil microorganisms decomposed organic substances (Persello-Cartieaux et al., 2003), accelerating nutrient turnover and increasing nutrient supply for plants (Zhu et al., 2017; Song et al., 2019). Our study revealed that moss growth parameters positively correlated with soil total nitrogen and soil organic matter, which negatively correlated with the relative abundance of Actinobacteria (Supplementary Figure 2). This could be because Actinobacteria members degrade complex compounds, such as polysaccharides and phenolic compounds, improving the nutritional and growth status of biocrusts (Vetrovsky et al., 2014). The richness and diversity of eukaryotes were not consistent with prokaryotes because eukaryotes are slow to respond to environmental changes and prokaryotes reproduce in large numbers to compete for nutrition and space resources. Proliferation of the dominant phylum Ascomycota inhibited the growth of other eukaryotes, reducing their overall number.

### Prediction of Metabolic Function in Moss Crusts Microbiome

Using PICRUSt2 to predict the metabolic function abundance of moss crust microorganisms could improve our understanding of the changes in microbial metabolic activity and strength under the influence of exogenous microorganisms. The results of the functional prediction were used to explain and support the differences in moss morphology and soil nutrients between the control and the best-performing treatment.

In this study, exogenous microorganisms enhanced the metabolic intensity of microorganisms in artificial moss crusts, consistent with Zhou et al. (2018), who found that exogenous microorganisms stimulate soil microorganism growth and improve the abundance and metabolic activity of microorganisms involved in soil metabolic pathways, optimizing the microecosystem. Our results showed that prokaryotes in the best-performing treatment used amino acids, such as L-isoleucine, L-valine, L-serine, glycine, and branched amino acids, as carbon and nitrogen sources to complete their growth and promote protein synthesis and transport (Figure 4 and Supplementary Table 6). In this sense, prokaryotes synthesize amino acids as plant growth regulators for moss growth (Schobert et al., 1988). Another important feature of eukaryotes in the best-performing treatment would be the high abundance of metabolic pathways associated with nucleotide biosynthesis, specifically adenosine nucleotides, pyrimidine deoxyribonucleotides, and guanosine nucleotides, indicating that these metabolic pathways are important for eukaryote reproduction (Figure 4 and Supplementary Table 6). Fatty acids are used directly by soil fungal communities as lipid precursors or carbon sources by β-oxidation, releasing large amounts of energy for fungal reproduction (Miralles et al., 2021).

According to the PICRUSt2 prediction, the carbon cycling process in soil co-occurs for prokaryotes and eukaryotes. Soil microbe functions related to carbon fixation and cellulose decomposition had relatively high abundance, while hemicellulose and lignin decomposition, nitrogen fixation, nitrification, and denitrification had relatively low abundance at each sampling time (Figure 5). This indicates that the microbes in artificial moss crusts might decompose cellulose well but be weak at decomposing hemicellulose and lignin and functions related to nitrogen cycling. On day 15, the relative abundance of C- and N-related enzymes differed between the best-performing treatment and control (Figure 5 and Supplementary Table 7). For instance, the best-performing treatment had significantly higher propionyl-CoA carboxylase than the control. It is the key enzyme involved in the 3-hydroxypropionic acid pathway of autotrophic CO_2_ fixation in the Calvin cycle, one of the most important drivers of the soil carbon cycle, favoring total organic carbon accumulation (Rubin-Blum et al., 2019). *Chlorella vulgaris* is a highly efficient photosynthetic microorganism, capable of carbon sequestration. Thus, we speculate that the added *Chlorella vulgaris* may be involved in the metabolic pathway of secreted propionyl-CoA carboxylase. Our results corroborate that the best-performing treatment enriched the moss crusts in a hydrolytic enzyme pool, including cellulase, endo-1,3(4)-β-glucanase, and β-glucuronidase involved in cellulose decomposition and catechol 1,2-dioxygenase involved in lignin decomposition. Cellulose and lignin decomposition may be due to the catabolism of the added *Streptomyces pactum* and Ascomycota, whose growth was stimulated by exogenous microorganisms. Other studies also support the premise that exogenous microbial agents promote soil carbon metabolism and improve the decomposition rates of total carbon, water-soluble carbon, cellulose, hemicellulose, and lignin (Yu et al., 2011).

The addition of *Streptomyces pactum* and *Chlorella vulgaris* enhanced the relative abundance of Ascomycota. *Chlorella vulgaris* improved the organic matter content in moss crust through photosynthesis. At the same time, *Streptomyces pactum* and Ascomycota degraded cellulose and lignin, decomposing organic matter and thus improving the soil environment and moss morphological indexes. In this study, the microbial community function and genes related to C and N metabolic processes and enzymes were preliminarily analyzed in PICRUSt2 using the taxonomic composition of the samples. Considering the limitations of PICRUSt2, the microbial community function should be further studied in combination with metagenomic sequencing technology.

## Conclusion

Our study revealed the potential role of exogenous microbes in moss inoculum propagation. Exogenous microorganisms promoted moss growth and aboveground biomass and increased eukaryotic community diversity and richness. They may also enhance soil microbial functional and metabolic diversity, such as growth and reproduction, carbon fixation, and cellulose and lignin decomposition. Adding exogenous microbes such as *Streptomyces pactum* and *Chlorella vulgaris* positively affected nutrient uptake, enhancing moss growth, and could be a cost-effective technology for promoting moss reproduction. Our study also offers a theoretical basis for the large-scale proliferation of moss inoculum.

## Data Availability Statement

The datasets presented in this study can be found in online repositories. The names of the repository/repositories and accession number(s) can be found below: https://www.ncbi.nlm.nih.gov/bioproject/PRJNA669273.

## Author Contributions

CB conceived the ideas and designed the methodology. CT, HW, XB, and YL collected the data. CT and HW analyzed the data and wrote the manuscript. SW and KS contributed critically to the drafts. All authors gave final approval for publication.

## Conflict of Interest

The authors declare that the research was conducted in the absence of any commercial or financial relationships that could be construed as a potential conflict of interest.

## Publisher’s Note

All claims expressed in this article are solely those of the authors and do not necessarily represent those of their affiliated organizations, or those of the publisher, the editors and the reviewers. Any product that may be evaluated in this article, or claim that may be made by its manufacturer, is not guaranteed or endorsed by the publisher.

## Footnotes

1
http://algae.ihb.ac.cn/

2
https://unite.ut.ee/repository.php
