# Supplementary material for: Exogenous Microorganisms Promote Moss Biocrust Growth by Regulating the Microbial Metabolic Pathway in Artificial Laboratory Cultivation
Source: Front Microbiol. 2022 Mar 2;13:819888. doi: 10.3389/fmicb.2022.819888 (PMC8924459; doi:10.3389/fmicb.2022.819888)
Supplement: Supplementary file 1 [file Data_Sheet_1.pdf]

Supplemental Figures

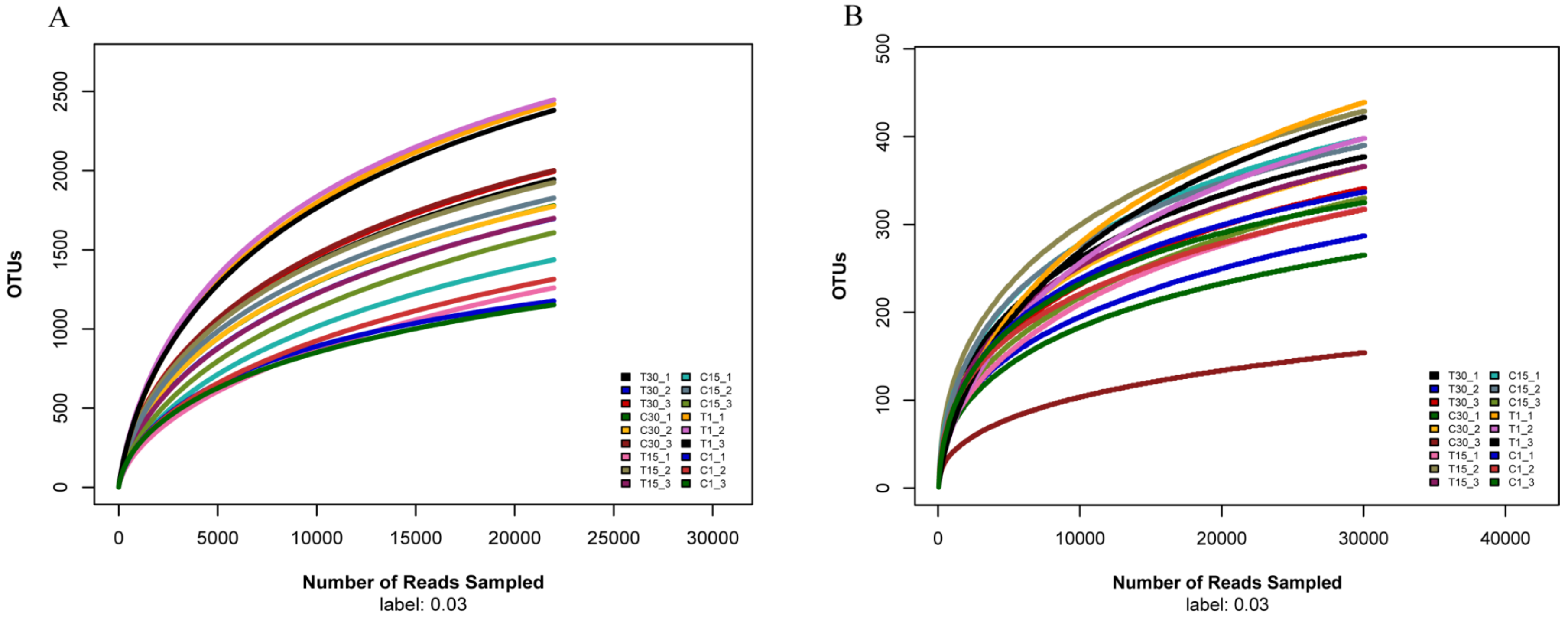

Supplementary Figure 1. Rarefaction curves of internal transcribed spacer DNA for high-throughput sequencing of prokaryote (A) and eukaryote (B) from soil with different biocrusts. T1: Treatment at Day 1; T15: Treatment at Day 15; T30: Treatment at Day 30; C1: Control at Day 1; C15: Control at Day 15; C30: Control at Day 30.

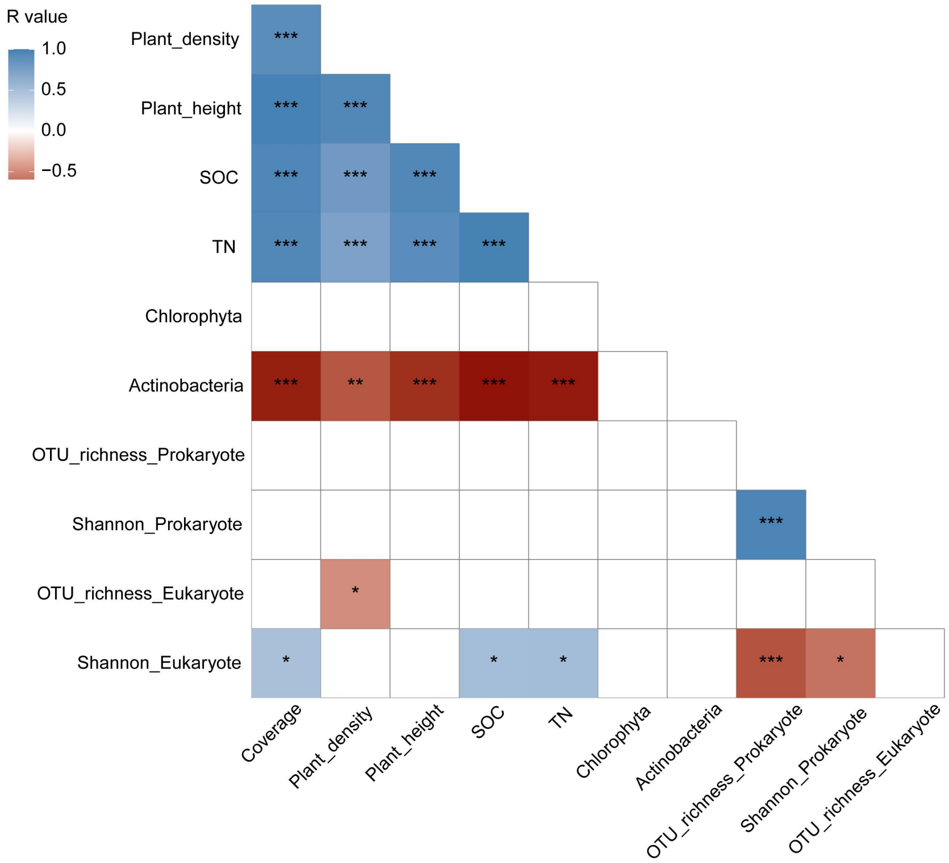

Supplementary Figure 2. Pearson correlation analyses were performed between microbial parameters, physicochemical variables and moss growth indexes. The blue and red colors indicate positive and negative correlations, respectively. Numbers in the figure indicate correlation coefficient. The significances of correlation analyses are marked with asterisks (*) at different significance levels (* for $P < 0.05$, ** for $P < 0.01$ and *** for $P < 0.001$).

Supplemental Tables

Supplemental Table1. The number of clean tags for 16S rRNA gene and 18S rRNA gene and the average percentage of the final tags that were used in analysis

Time	Treatment	Samples	16S rRNA gene			18S rRNA gene		
			Clean_tags	Final_tags	Percentage	Clean_tags	Final_tags	Percentage
1d	CK	CK_1	50048	21977	43.9%	68794	29136	42.4%
		CK_2	55538	21977	39.6%	147781	29136	19.7%
		CK_3	61647	21977	35.6%	106289	29136	27.4%
	SC	SC_1	40992	21977	53.6%	36051	29136	80.8%
		SC_2	56223	21977	39.1%	39337	29136	74.1%
		SC_3	67447	21977	32.6%	34716	29136	83.9%
15d	CK	CK_1	101388	21977	21.7%	230881	29136	12.6%
		CK_2	96478	21977	22.8%	63419	29136	45.9%
		CK_3	78486	21977	28.0%	65381	29136	44.6%
	SC	SC_1	24444	21977	89.9%	52829	29136	55.2%
		SC_2	39424	21977	55.7%	89223	29136	32.7%
		SC_3	60428	21977	36.4%	68364	29136	42.6%
30d	CK	CK_1	67051	21977	32.8%	44142	29136	66.0%
		CK_2	57641	21977	38.1%	188470	29136	15.5%
		CK_3	56714	21977	38.8%	29535	29136	98.6%
	SC	SC_1	460178	21977	4.8%	32700	29136	89.1%
		SC_2	72092	21977	30.5%	82475	29136	35.3%
		SC_3	116198	21977	18.9%	142337	29136	20.5%

Supplemental Table 2. The morphological changes in the artificial moss crusts

Treatment	Samples	Coverage/%					Plant density/shoots·cm^{-2}					Plant height/mm				
		7d	15d	22d	30d	Average	7d	15d	22d	30d	Average	7d	15d	22d	30d	Average
ABC	ABC_1	20.08	60.00	89.13	92.86	90.95(b)	3.09	6.21	14.03	27.07	23.73(a)	0.24	1.10	2.14	2.02	1.97(a)
	ABC_2	22.72	61.43	87.94	91.43		2.61	7.04	14.16	23.25		0.24	1.08	1.86	1.93	
	ABC_3	21.32	64.29	84.00	88.57		2.79	6.75	15.97	20.88		0.26	1.07	2.12	1.97	
ASM	ASM_1	20.82	54.29	90.92	91.43	92.38(b)	4.09	9.49	13.36	24.21	23.94(a)	0.19	1.04	1.67	2.12	1.92(a)
	ASM_2	20.90	54.29	92.14	94.29		3.10	8.37	16.85	25.97		0.21	1.40	1.56	2.09	
	ASM_3	19.42	65.71	93.52	91.43		2.69	8.13	11.52	21.63		0.18	1.11	1.32	1.56	
BM	BM_1	19.12	60.00	88.18	90.00	90.48(b)	2.63	7.07	10.45	25.63	24.78(a)	0.13	1.05	1.46	2.07	2.24(a)
	BM_2	21.07	55.71	92.19	88.57		2.46	8.35	12.00	22.99		0.13	1.07	1.78	1.97	
	BM_3	19.16	61.43	89.92	92.86		2.41	5.73	13.84	25.71		0.11	1.19	1.74	2.67	
SC	SC_1	22.99	71.43	91.32	95.71	97.14(a)	3.04	7.76	14.77	25.07	28.31(a)	0.20	1.08	1.89	2.25	2.28(a)
	SC_2	22.83	74.29	92.29	97.14		2.84	8.35	17.55	26.00		0.21	1.41	1.91	2.12	
	SC_3	21.57	65.71	90.95	98.57		3.04	7.57	18.05	33.87		0.27	1.60	1.42	2.46	
CK	CK_1	18.42	54.86	87.55	89.29	89.48(b)	3.55	11.04	15.12	26.11	26.86(a)	0.23	1.07	1.55	1.97	2.08(a)
	CK_2	17.20	56.43	86.87	89.29		3.21	7.92	18.13	27.19		0.21	1.55	1.83	2.17	
	CK_3	16.76	57.71	85.67	89.86		1.96	5.63	17.76	27.29		0.23	1.03	2.03	2.10	

Supplemental Table 3. The physicochemical characterisation in the moss crusts at three moss development stages

Time	Treatment	SOC（$g \cdot kg^{-1}$）	TN($g \cdot kg^{-1}$)
1d	CK	2.97(a)	0.13(a)
	SC	3.34(a)	0.15(a)
15d	CK	14.32(a)	0.68(a)
	SC	13.20(a)	0.67(a)
30d	CK	16.46(a)	0.72(a)
	SC	14.34(b)	0.72(a)

Notes: The data represent the mean values (n = 3). T-tests shows the significance among groups at α=0.05 level.

Supplemental Table 4. T-test of microbial relative abundance (%) at phylum level in biocrusts

Taxonomies		Days					
		1d		15d		30d	
	Phylum	Treatment	Control	Treatment	Control	Treatment	Control
Prokaryote	Proteobacteria	24.0	31.6	57.3	61.7	48.9	48.7
	Actinobacteria	28.2	38.7	6.4	6.6	6.1	5.3
	Bacteroidetes	3.3	18.8	9.0	4.7	7.8	7.8
	Cyanobacteria	4.7	0.7	10.6	9.5	8.8	9.5
	Firmicutes	0.4	1.2	5.0	7.2	9.5	9.9
	Chloroflexi	9.7	1.9	3.8	2.7	6.2	6.9
	Planctomycetes	7.8	0.9	4.6	4.5	5.7	4.8
	Acidobacteria	13.2	1.0	1.4	1.3	2.1	2.2
	Gemmatimonadetes	2.9	1.4	0.4	0.5	1.3	1.6
	Thaumarchaeota	3.5	0.3	0.2	0.3	0.7	0.5
	Others	2.3	3.5	1.3	1.0	2.9	2.8
Eukaryote	Ascomycota	10.3	61.2	48.4	45.3	51.6	40.8
	Streptophyta	85.3	3.3	12.3	9.5	17.4	10.2
	Others	2.3	4.1	31.1	36.5	18.8	37.1
	Chytridiomycota	0.2	3.6	3.5	3.7	10.2	8.0
	Basidiomycota	0.9	22.6	0.8	0.3	0.6	0.4
	Chlorophyta	1.0	5.1	2.6	3.2	0.9	2.9
	Mucoromycota	0.0	0.0	1.0	0.9	0.2	0.2
	Eustigmatophyceae	0.0	0.0	0.2	0.3	0.0	0.3
	Blastocladiomycota	0.0	0.0	0.1	0.1	0.1	0.0
	Apicomplexa	0.0	0.0	0.1	0.1	0.1	0.0
	Rotifera	0.0	0.2	0.0	0.0	0.0	0.0

Supplemental Table 5. Diversity indexes of soil samples with different development stages

Time	Treatment	Samples	Prokaryote									
			OTU number	Average (ANOVA)	Chao1	Average (ANOVA)	ACE	Average (ANOVA)	Shannon	Average (ANOVA)	Simpson	Coverage	PD_whole_tree
1d	CK	CK_1	1177	1214(b)	1513.6839	1693.24(b)	1512.02	1728.46(b)	6.8236004	6.96(b)	0.955896	0.9835737	89.80141
		CK_2	1314		1970.933		2065.99		6.9895002		0.9640037	0.9752924	96.84738
		CK_3	1152		1595.0939		1607.35		7.0623811		0.9689064	0.9817537	93.99602
	SC	SC_1	2421	2416(a)	3107.875	3163.43(a)	3181.31	3182.85(a)	9.140816	9.26(a)	0.9921017	0.9642808	158.44917
		SC_2	2447		3212.3409		3189.34		9.3968739		0.9953098	0.9644173	160.49248
		SC_3	2381		3170.0697		3177.90		9.2357282		0.9940983	0.9637348	156.22971
15d	CK	CK_1	1437	1624(a)	2133.2397	2396.81(a)	2170.75	2399.47(a)	7.1157085	7.59(a)	0.9751863	0.9735633	107.89838
		CK_2	1827		2585.723		2540.52		8.503678		0.989876	0.970423	124.29377

									4		8	6	
		CK_3	1608		2471.4806		2487.13		7.154307		0.9618925	0.9696046	120.89144
	SC	SC_1	1260	1628(a)	2067.1582	2364.46(a)	2083.37	2399.98(a)	6.5905759	7.69(a)	0.9603654	0.9743823	100.05688
		SC_2	1925		2547.4503		2627.44		8.7231237		0.9931861	0.9702871	142.36222
		SC_3	1699		2478.7729		2489.12		7.7629959		0.9725785	0.9702871	116.39642
30d	CK	CK_1	1779	1852(a)	2587.7179	2588.26(a)	2550.41	2585.76(a)	8.3485823	8.43(a)	0.9904124	0.9697411	124.286
		CK_2	1775		2491.0638		2484.75		8.469459		0.9923302	0.9710607	128.22577
		CK_3	2001		2686		2722.13		8.4774248		0.9857799	0.9687855	141.07174
	SC	SC_1	1944	1879(a)	2705.875	2623.55(a)	2689.46	2645.94(a)	8.7174756	8.44(a)	0.9923657	0.9686035	144.97327
		SC_2	1697		2388.5411		2451.14		7.957689		0.9838571	0.9710607	127.77129
		SC_3	1995		2776.2222		2797.22		8.6506979		0.9887896	0.9676025	143.94966

Time	Treatment	Samples	Eukaryote										
			OTU number	Average (ANOVA)	Chao1	Average (ANOVA)	ACE	Average (ANOVA)	Shannon		Simpson	Coverage	PD_whole_tree
1d	CK	CK_1	337		424.20833		424.03924		4.3744263		0.8898133	0.9968424	50.50075
		CK_2	317	326(b)	425.02273	413.65(b)	434.82276	421.05(b)	4.4706957	4.34(a)	0.8885454	0.9966365	43.10301
		CK_3	325		391.72549		404.29992		4.164005		0.8777769	0.9971513	45.78862
	SC	SC_1	439		578.95122		612.2144		1.7284909		0.3002306	0.9947831	30.94419
		SC_2	398	420 (a)	502.50633	550.87(a)	519.73931	567.06(a)	1.7286133	1.74(b)	0.306748	0.9955725	27.93997
		SC_3	422		571.14286		569.21491		1.7685527		0.3161739	0.9950233	28.86616
15d	CK	CK_1	398	373(a)	493.15873	474.23(a)	510.35213	493.12(a)	5.0989604	4.80(a)	0.9341848	0.9962246	29.2911
		CK_2	390		482.34483		489.8273		4.9572686		0.9350756	0.9964305	29.46038

		CK_3	330		447.18519		479.1919		4.3344173		0.8969403	0.9961216	25.95577
	SC	SC_1	318	371(a)	397.83871	467.09(a)	419.76565	487.04(a)	4.5234809	4.73(a)	0.9202154	0.9965678	24.39213
		SC_2	429		544.2381		558.70149		5.2026295		0.9367263	0.9958471	29.37914
		SC_3	366		459.19355		482.65556		4.4609932		0.8929285	0.9962932	26.32728
30d	CK	CK_1	265	262(a)	355	360.73(a)	350.88828	359.44(a)	4.0828212	3.72(a)	0.869794	0.9972199	21.95139
		CK_2	366		506.87755		507.44168		4.2484504		0.8594399	0.99595	25.82755
		CK_3	154		220.3		219.99509		2.8275192		0.7489139	0.9982153	16.45145
	SC	SC_1	377	335(a)	463.3871	438.74(a)	482.6008	450.16(a)	4.8112613	4.39(b)	0.9174647	0.9964305	27.15425
		SC_2	287		395.90244		402.07539		4.4485429		0.9024666	0.9967394	23.62946
		SC_3	341		456.9375		465.79992		3.9071859		0.808862	0.9963619	28.18404

Notes: Average shows the mean value of OTUs and Shannon indexes of related biocrusts. ANOVA (ANalysis Of Variance) shows the significance among groups at α=0.05 level using the Duncan method.

Supplemental Table 6. PICRUSt2 function prediction results in abundance of metabolic pathways during moss development for prokaryote and eukaryote with the best performing exogenous microbes and control.

Taxonomies	Metabolic pathways	Days					
		1d		15d		30d	
		Treatment	Control	Treatment	Control	Treatment	Control
Prokaryote	aerobic respiration I (cytochrome c)	1.38(b)	1.64(a)	1.81(a)	1.49(b)	1.42(a)	1.38(a)
	pyruvate fermentation to isobutanol (engineered)	1.10(a)	1.01(b)	1.15(a)	1.02(b)	0.98(a)	1.06(a)
	L-isoleucine biosynthesis II	0.89(b)	0.96(a)	1.06(a)	0.94(b)	0.93(a)	0.93(a)
	L-isoleucine biosynthesis I (from threonine)	0.87(b)	0.94(a)	1.05(a)	0.91(b)	0.89(a)	0.92(a)
	L-valine biosynthesis	0.87(b)	0.94(a)	1.05(a)	0.91(b)	0.89(a)	0.92(a)
	cis-vaccenate biosynthesis	0.84(a)	0.61(b)	0.82(b)	0.89(a)	0.86(a)	0.85(a)
	gondoate biosynthesis (anaerobic)	0.83(a)	0.58(b)	0.78(b)	0.88(a)	0.85(a)	0.84(a)
	superpathway of branched amino acid biosynthesis	0.73(b)	0.79(a)	0.85(a)	0.77(b)	0.76(a)	0.75(a)
	fatty acid elongation-saturated	0.77(a)	0.58(b)	0.75(b)	0.80(a)	0.77(a)	0.78(a)
	fatty acid salvage	0.67(b)	0.79(a)	0.75(a)	0.62(b)	0.66(a)	0.67(a)
	urate biosynthesis/inosine 5'-phosphate degradation	0.65(b)	0.87(a)	0.71(a)	0.64(b)	0.65(a)	0.64(a)
	pentose phosphate pathway (non-oxidative branch)	0.67(a)	0.69(a)	0.72(a)	0.67(b)	0.69(a)	0.70(a)

	L-isoleucine biosynthesis III	0.65(b)	0.71(a)	0.73(a)	0.68(b)	0.67(a)	0.65(a)
	TCA cycle I (prokaryotic)	0.65(b)	0.70(a)	0.70(a)	0.67(b)	0.65(a)	0.64(a)
	superpathway of L-serine and glycine biosynthesis I	0.64(a)	0.60(a)	0.70(a)	0.64(b)	0.67(a)	0.65(a)
	fatty acid; beta-oxidation I	0.64(a)	0.73(a)	0.68(a)	0.60(a)	0.62(a)	0.64(a)
	CDP-diacylglycerol biosynthesis I	0.63(b)	0.65(a)	0.63(b)	0.66(a)	0.68(a)	0.64(a)
	CDP-diacylglycerol biosynthesis II	0.63(b)	0.65(a)	0.63(b)	0.66(a)	0.68(a)	0.64(a)
	TCA cycle V (2-oxoglutarate: ferredoxin oxidoreductase)	0.61(b)	0.66(a)	0.70(a)	0.64(b)	0.63(a)	0.60(b)
	superpathway of L-isoleucine biosynthesis I	0.58(b)	0.64(a)	0.66(a)	0.62(b)	0.62(a)	0.60(a)
	superpathway of pyrimidine nucleobases salvage	0.58(b)	0.66(a)	0.63(a)	0.61(b)	0.60(a)	0.58(a)
	superpathway of adenosine nucleotides de novo biosynthesis I	0.57(b)	0.64(a)	0.66(a)	0.61(b)	0.59(a)	0.57(a)
Eukaryote	nicotine degradation IV	5.14(a)	6.48(a)	3.02(a)	4.34(a)	4.76(a)	6.70(a)
	phytol degradation	4.38(a)	4.71(a)	3.75(b)	4.17(a)	4.43(a)	4.45(a)
	aerobic respiration I	3.36(a)	2.80(a)	4.79(a)	3.92(b)	3.81(a)	3.09(a)
	aerobic respiration II	3.36(a)	2.80(a)	4.79(a)	3.92(b)	3.81(a)	3.09(a)
	fatty acid; beta-oxidation II	2.24(a)	2.01(a)	3.02(a)	2.27(b)	2.20(a)	2.06(a)
	fatty acid; beta-oxidation	2.03(a)	1.61(b)	2.89(a)	2.11(b)	2.01(a)	1.83(a)

	fatty acid; beta-oxidation VI	1.91(a)	1.91(a)	1.38(b)	1.91(a)	1.92(a)	1.81(a)
	unsaturated, even numbered fatty acid; beta-oxidation	1.63(a)	1.46(a)	1.99(a)	1.68(b)	1.71(a)	1.60(a)
	fatty acid; beta-oxidation I	1.53(b)	2.06(a)	0.70(b)	1.50(a)	1.57(a)	1.55(a)
	fatty acid elongation-saturated	1.69(a)	1.87(a)	0.58(a)	1.07(a)	0.79(a)	2.28(a)
	palmitate biosynthesis I	1.67(a)	1.86(a)	0.57(a)	1.05(a)	0.78(a)	2.23(a)
	adenosine ribonucleotides de novo biosynthesis	1.62(a)	1.15(a)	1.72(a)	1.62(a)	1.45(a)	1.28(a)
	4-hydroxybenzoate biosynthesis V	1.44(b)	2.05(a)	0.61(b)	1.38(a)	1.45(a)	1.48(a)
	glyoxylate cycle	1.37(a)	1.33(a)	1.48(a)	1.46(a)	1.48(a)	1.41(a)
	GDP-mannose biosynthesis	1.33(a)	1.37(a)	1.54(a)	1.42(a)	1.43(a)	1.34(a)
	D-myo-inositol (1,4,5)-trisphosphate biosynthesis	1.31(a)	1.23(a)	1.38(a)	1.37(a)	1.39(a)	1.31(a)
	TCA cycle II	1.29(a)	1.14(a)	1.64(a)	1.40(a)	1.37(a)	1.24(a)
	TCA cycle III	1.28(a)	1.09(a)	1.58(a)	1.40(a)	1.38(a)	1.23(a)
	adenosine nucleotides de novo biosynthesis II	1.30(a)	1.07(a)	1.68(a)	1.37(b)	1.35(a)	1.18(a)
	tRNA charging	1.27(a)	1.08(a)	1.60(a)	1.38(a)	1.34(a)	1.21(a)
	adenosine nucleotides de novo biosynthesis I	1.28(a)	1.08(a)	1.63(a)	1.36(b)	1.31(a)	1.16(a)
	glyoxylate cycle and fatty acid degradation	1.32(a)	1.27(a)	0.96(b)	1.35(a)	1.37(a)	1.26(a)
	guanosine nucleotides degradation II	1.17(a)	1.16(a)	1.35(a)	1.28(a)	1.33(a)	1.16(a)

	acetyl-CoA biosynthesis	1.21(a)	1.11(a)	1.44(a)	1.26(a)	1.26(a)	1.19(a)
	pyrimidine deoxyribonucleotides de novo biosynthesis I	1.17(a)	0.97(a)	1.63(a)	1.24(b)	1.25(a)	1.08(a)
	guanosine nucleotides degradation	1.11(a)	1.11(a)	1.29(a)	1.24(a)	1.29(a)	1.10(a)
	L-phenylalanine degradation IV	1.12(a)	0.90(a)	1.39(a)	1.18(b)	1.18(a)	1.16(a)
	pyruvate fermentation to isobutanol	1.04(a)	1.12(a)	1.29(a)	1.14(b)	1.17(a)	1.05(a)
	guanosine nucleotides de novo biosynthesis I	1.09(a)	0.91(b)	1.45(a)	1.17(b)	1.20(a)	1.04(a)
	methyl ketone biosynthesis	1.15(b)	1.41(a)	0.51(b)	1.09(a)	1.14(a)	1.13(a)
	pentose phosphate pathway	1.02(a)	1.18(a)	1.17(a)	1.03(a)	1.04(a)	1.01(a)
	pyrimidine deoxyribonucleotide phosphorylation	1.05(a)	0.84(a)	1.49(a)	1.10(b)	1.11(a)	0.96(a)
	pyrimidine nucleobases salvage	1.03(a)	0.88(a)	1.41(a)	1.09(b)	1.09(a)	0.97(a)
	L-serine and glycine biosynthesis I	0.97(a)	0.99(a)	1.27(a)	1.02(b)	1.04(a)	0.98(a)
	L-valine biosynthesis	0.95(a)	1.02(a)	1.14(a)	1.04(b)	1.06(a)	0.97(a)

Notes: The data represent the mean values (n = 3). T-tests shows the significance among groups at α=0.05 level.

Supplemental Table 7. PICRUSt2 function prediction results in the abundance of enzymes during moss development for prokaryote and eukaryote with the best performing exogenous microbes and control.

Taxonomies	Enzymes (x 10^{-4})	Days					
		1d		15d		30d	
		Treatment	Control	Treatment	Control	Treatment	Control
Prokaryote	4-hydroxybutanoyl-CoA dehydratase	7.36(a)	3.94(a)	39.82(a)	8.93(a)	11.46(a)	4.73(a)
	Pyruvate carboxylase	352.63(b)	487.86(a)	348.52(a)	381.07(a)	380.41(a)	287.18(a)
	Acetyl-CoA carboxylase	3304.40(b)	3477.55(a)	3737.60(a)	3549.60(a)	3455.79(a)	3331.48(a)
	Propionyl-CoA carboxylase	1246.70(b)	2292.71(a)	1914.99(a)	1378.84(b)	1334.93(a)	1260.31(a)
	Carbon-monoxide dehydrogenase (ferredoxin)	18.38(a)	0.65(a)	0.78(a)	19.95(a)	50.88(a)	26.96(a)
	Ribulose-bisphosphate carboxylase	471.76(a)	134.43(b)	322.66(a)	422.98(a)	508.53(a)	473.46(a)
	Cellulase	938.67(a)	930.24(a)	956.11(a)	994.00(a)	1115.02(a)	1015.57(a)
	Endo-1,3(4)-beta-glucanase	1.68(a)	0.72(a)	8.89(a)	2.56(b)	4.72(a)	2.04(a)
	Beta-glucuronidase	66.13(a)	67.88(a)	143.76(a)	70.64(b)	74.58(a)	57.77(b)
	Xylan 1,4-beta-xylosidase	203.84(a)	95.20(b)	73.96(b)	232.06(a)	214.69(a)	192.78(a)
	Endo-1,4-beta-xylanase	262.39(b)	351.51(a)	294.96(a)	306.55(a)	346.59(a)	288.75(a)
	Catechol 1,2-dioxygenase	128.14(a)	59.50(a)	138.05(a)	84.22(b)	86.78(a)	118.00(a)

	Nitrogenase	363.21(a)	86.77(a)	189.79(a)	333.19(a)	394.86(a)	414.07(a)
	Ammonia monooxygenase	6.22(a)	4.33(a)	42.23(a)	11.05(a)	12.04(a)	5.40(a)
	Nitrite reductase (NO-forming)	115.41(a)	80.94(a)	173.54(a)	140.14(a)	167.97(a)	129.68(a)
Eukaryote	Pyruvate carboxylase	744.34(b)	1007.60(a)	882.44(a)	743.78(a)	770.61(a)	743.99(a)
	Acetyl-CoA carboxylase	0.23(a)	0.11(a)	0.00(a)	0.23(a)	0.02(a)	0.02(a)
	Propionyl-CoA carboxylase	432.13(a)	484.73(a)	780.11(a)	454.34(b)	459.41(a)	358.76(a)
	Cellulase	616.79(a)	356.17(a)	761.26(a)	622.17(b)	491.66(a)	561.00(a)
	Endo-1,3(4)-beta-glucanase	123.77(a)	36.37(a)	15.72(a)	71.34(a)	31.29(a)	96.10(a)
	Beta-glucuronidase	604.82(b)	914.66(a)	195.08(b)	567.62(a)	609.53(a)	653.17(a)
	Xylan 1,4-beta-xylosidase	0.46(a)	120.91(a)	2.71(a)	0.14(a)	2.71(a)	0.78(a)
	Endo-1,4-beta-xylanase	22.48(a)	0.56(a)	1.68(a)	12.81(a)	4.97(a)	2.74(a)
	Catechol 1,2-dioxygenase	79.14(a)	35.82(a)	14.24(a)	45.79(a)	26.89(a)	91.46(a)

Notes: The data represent the mean values (n = 3). T-tests shows the significance among groups at α=0.05 level.
